# Versatile software and hardware combo enabling photon counting acquisition and real-time display for multiplexing, 2D and continuous 3D two-photon imaging applications

**DOI:** 10.1117/1.NPh.9.3.031920

**Published:** 2022-09-20

**Authors:** Hagai Har-Gil, Lior Golgher, David Kain, Pablo Blinder

**Affiliations:** a Tel Aviv University, Sagol School of Neuroscience, Tel Aviv, Israel; b Tel Aviv University, Department of Neurobiology, George S. Wise Faculty of Life Sciences, Tel Aviv, Israel

**Keywords:** optics, photonics, photon counting, rendering, two-photon imaging

## Abstract

**Significance:**

rPySight brings a flexible and highly customizable open-software platform built around a powerful multichannel digitizer; combined, it enables performing complex photon counting-based experiments. We exploited advanced programming technology to share the photon counting stream with the graphical processing unit (GPU), making possible real-time display of two-dimensional (2D) and three-dimensional (3D) experiments and paving the road for other real-time applications.

**Aim:**

Photon counting improves multiphoton imaging by providing better signal-to-noise ratio in photon-deprived applications and is becoming more widely implemented, as indicated by its increasing presence in many microscopy vendor portfolios. Despite the relatively easy access to this technology offered in commercial systems, these remain limited to one or two channels of data and might not enable highly tailored experiments, forcing most researchers to develop their own electronics and code. We set to develop a flexible and open-source interface to a cutting-edge multichannel fast digitizer that can be easily integrated into existing imaging systems.

**Approach:**

We selected an advanced multichannel digitizer capable of generating 70M tags/s and wrote an open software application, based on Rust and Python languages, to share the stream of detected events with the GPU, enabling real-time data processing.

**Results:**

rPySight functionality was showcased in real-time monitoring of 2D imaging, improved calcium imaging, multiplexing, and 3D imaging through a varifocal lens. We provide a detailed protocol for implementing out-of-the-box rPySight and its related hardware.

**Conclusions:**

Applying photon-counting approaches is becoming a fundamental component in recent technical developments that push well beyond existing acquisition speed limitations of classical multiphoton approaches. Given the performance of rPySight, we foresee its use to capture, among others, the joint dynamics of hundreds (if not thousands) of neuronal and vascular elements across volumes, as is likely required to uncover in a much broader sense the hemodynamic transform function.

## 1 Introduction

Cutting-edge neuroscientific research has always been very demanding with respect to the tools it requires. In the field of *in vivo* microscopy, for example, imaging more neurons with better contrast will always create a higher degree of confidence in the results regardless of the specific question being asked. Thus, many researchers focus their efforts at improving the experimental apparatuses available to the imaging community with the hope that it will empower them and other groups to answer a variety of questions that were too ambitious to attack with previous-generation tooling.

Improvements are coming at a rapid pace and in all aspects concerning imaging experiments. A prime example is the integration of optical and optomechanical techniques to greatly extend the field of view (FOV) of two-photon microscope (2PM) without sacrificing optical performance in general, and image resolution in particular.^1^^–^^6^ Another important trend is the addition of adaptive optics into the 2PM optical path to modify the laser’s wavefront for specific purposes, such as improving resolution and contrast deep inside the sample^7^ or imaging more than one plane at once.^8^^,^^9^ In addition, software-focused innovation has also been crucial in the effort to improve imaging conditions. Examples include time-tested microscope-operating software,^10^ post-processing applications, libraries aimed at analyzing calcium signals,^11^^,^^12^ or libraries that infer spike timings.^13^

Finally, another enhancement that was proposed and implemented concerns the acquisition devices, and especially the use of photon counting, to reduce noise sources in the acquisition hardware and consequently improve the signal-to-noise ratio (SNR). In time-tagged photon counting, the measured signal is thresholded or discriminated from a continuous stream of relatively noisy voltage fluctuations to a list of discrete photon arrival times that more accurately represents the underlying quantized photon stream that arrived at the detector. The noisy original stream—a consequence of the ultra-sensitive detectors used in 2PM—is filtered, and the resulting data stream can either be digitized normally or converted into a list of photon arrival times for more advanced imaging modalities. This digital imaging modality stands in contrast to the standard analog integration modality, in which the raw voltage fluctuations from the photodetectors are sampled using analog-to-digital converters to a digital value. Photon counting has been shown both theoretically^14^^,^^15^ and experimentally^16^ to improve imaging conditions in a variety of applications. By utilizing digital acquisition using fast digitizers and relying only on the list of photon arrival times, our group imaged continuous three-dimensional (3D) volumes of mice neocortex at high frame rates.^17^

The work presented here expands on our efforts in this context by addressing the main downside of our previously-published application (PySight^17^), namely that it did not allow its users to see real-time images and volumes or multiplexed acquisitions, mandating instead a post-processing step. To mitigate this downside, we present *rPySight* and add-on solution to existing 2PM, which can render 2D and 3D images and volumes of photon counting data in real time, independently of our previous work.^17^ rPySight, an open source application, integrates with a fast digitizer (TimeTagger Ultra [TT], Swabian Instruments) that discriminates the photon stream from two channels and coordinates their data streams with synchronization signals from the scanning mirrors, generating images with qualitatively improved SNRs, which we quantify indirectly by showing improved performance in calcium imaging acquisition, a far more useful and better defined comparison. Importantly, the device supports a total of 18 channels that can be digitized simultaneously, opening the ability to perform complex experiments.

## 2 Materials and Methods

### 2.1 Animal Models

All animal-related work was done in accordance with the Tel Aviv University ethics committee for animal use and welfare, which adheres to standards established by the National Health Institute, Institutional Animal Care and Use Committee. A full description of the animal preparations procedure is detailed in Appendix A.

### 2.2 System Architecture

**Fig. 1:**
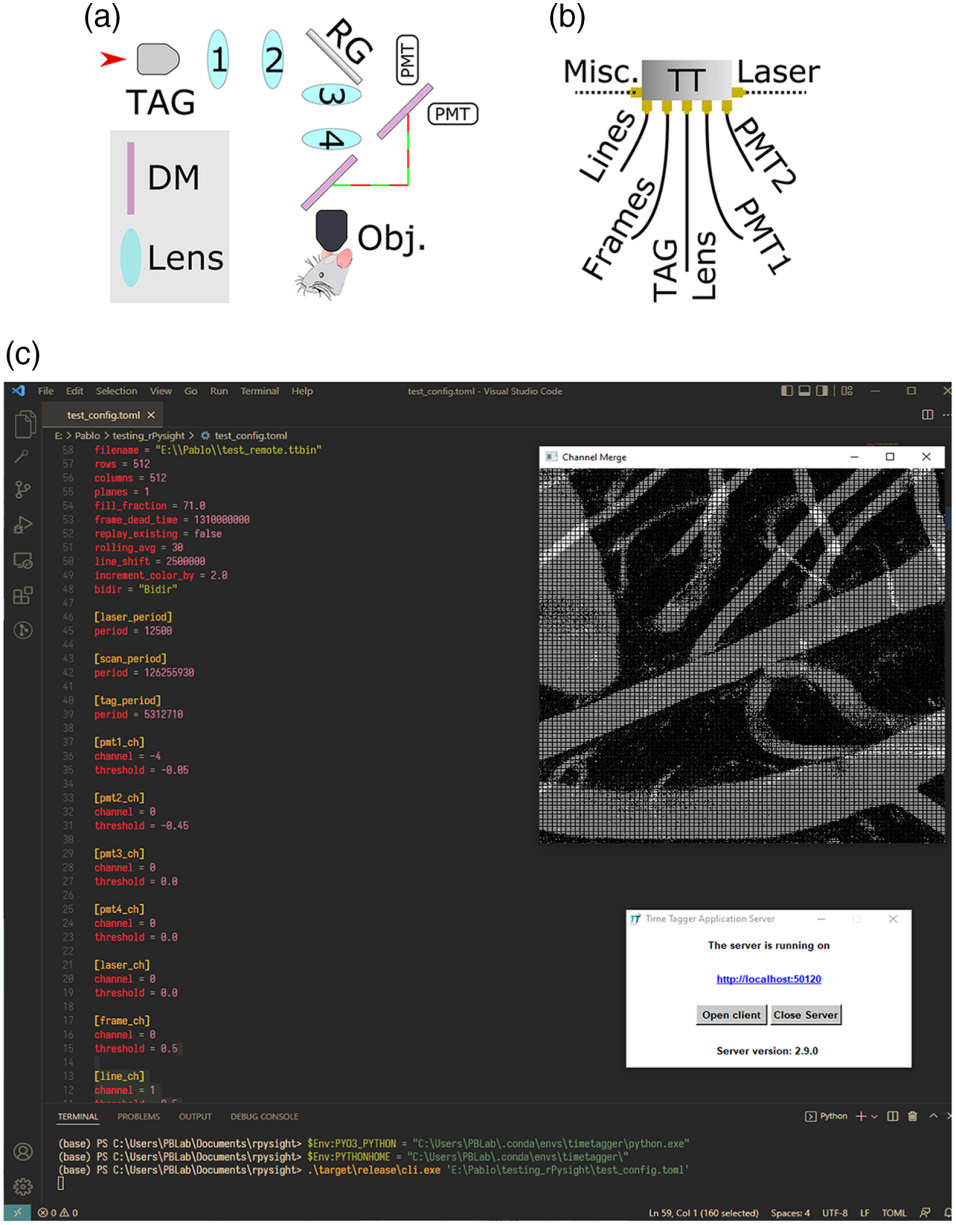
The optical, electronic, and software setup used for online photon counting. (a) Before passing through the objective, the input beam traverses through the TAG lens (when imaging in 3D) and its relay system, consisting of two lenses L1=45  mm and L2=60  mm. The beam bounces off the resonant-galvo scanners into the scan lens and tube lens, marked as L3 and L4, respectively. The emitted photons are collected through the objective and are routed using a dichroic mirror into the collection path, where a second dichroic mirror splits them into two spectral channels. (b) The TT wiring diagram. To build frames, rPySight requires either a line synchronization signal or a start of frame (but not both) and at least a single spectral channel to display images. Additional inputs such as the laser pulse synchronization, TAG lens phase signal, or others may be added as needed (up to 18 channels in total). (c) A screenshot showing pial blood vessels in an anesthetized mouse being imaged with rPySight. The key components are the text editor used to create or edit the configuration file, a command window and the TimeTagger server. Here, we used Visual Studio (Microsoft Inc.) both as editor and command window. In this particular case, we acquired an image of 512×512  pixels, at a frame rate of 30 fps. rPySight was set to display an average of 30 frames. Not shown in the image are the microscope control panels (ScanImage 2021, Vidrio Technologies, LCC). rPySight is agnostic to the software being used for microscope control as long as the required signals can be connected to the fast digitizer. RG, resonant-galvo scanner; DM, dichroic mirror; Obj., objective lens; PMT, photomultiplier tube (preamplifiers not shown); TAG, tunable acoustic gradient index of the refraction lens.

The optical setup of the proposed system is depicted in Fig. 1 and is detailed in Appendix B. The optical path as seen in Fig. 1(a) is a standard 2PM path with the (optional) tunable acoustic gradient (TAG) lens module added before the scanning mirrors, which enables 3D imaging. For standard 2D imaging, the basic configuration of the path should be kept. The photons that fluoresce from the sample are collected by H10770PA-40SEL photomultiplier tubes (PMT), which are connected via fast preamplifiers to the TT. Photon counting permits setting the PMT gains at much higher values than in analog acquisition due to the built-in filtration step in the form of the discriminator. From a practical point of view, in our basic analog setup, we use gain values of about $4\times 10^{5}$, whereas for digital acquisition we can ramp up the gain to $2\times 10^{6}$, a fivefold increase, and benefit from better photon detection rates.

Figure 1(b) shows the electronic connections to the digitizer. The TT receives inputs into a set of channels pre-defined in the rPySight configuration file. Each input is discriminated and digitized on the fly and sent via a USB 3.0 interface to the computer. The TT has potentially up to 18 input channels, which permits experimenters to record additional data streams such as transistor-transistor logic (TTL) signals for synchronization with other experimental apparatus, such information can be recorded to disk, although it remains unused by rPySight. The data exist in binary form in the same folder of the rPySight streams. A screenshot of an acquisition session using rPySight [Fig. 1(c)] shows blood vessels at the pial level of an anesthetized mice as well as the key software tools needed to run: a text editor to create or modify the configuration file, a console to issue commands, and the TT server that runs in the background.

### 2.3 Software

rPySight is an open-source application that we made available on GitHub^18^ licensed under the GNU Public License v3.0. Most of rPySight is written in the Rust programming language, while the parts that interact with the TT application programming interface are written in Python. rPySight is currently tested on Windows machines only but should work on UNIX-like operating systems assuming that the rest of the components of the microscope, such as the scanners and the TT, are working as well.

**Fig. 2:**
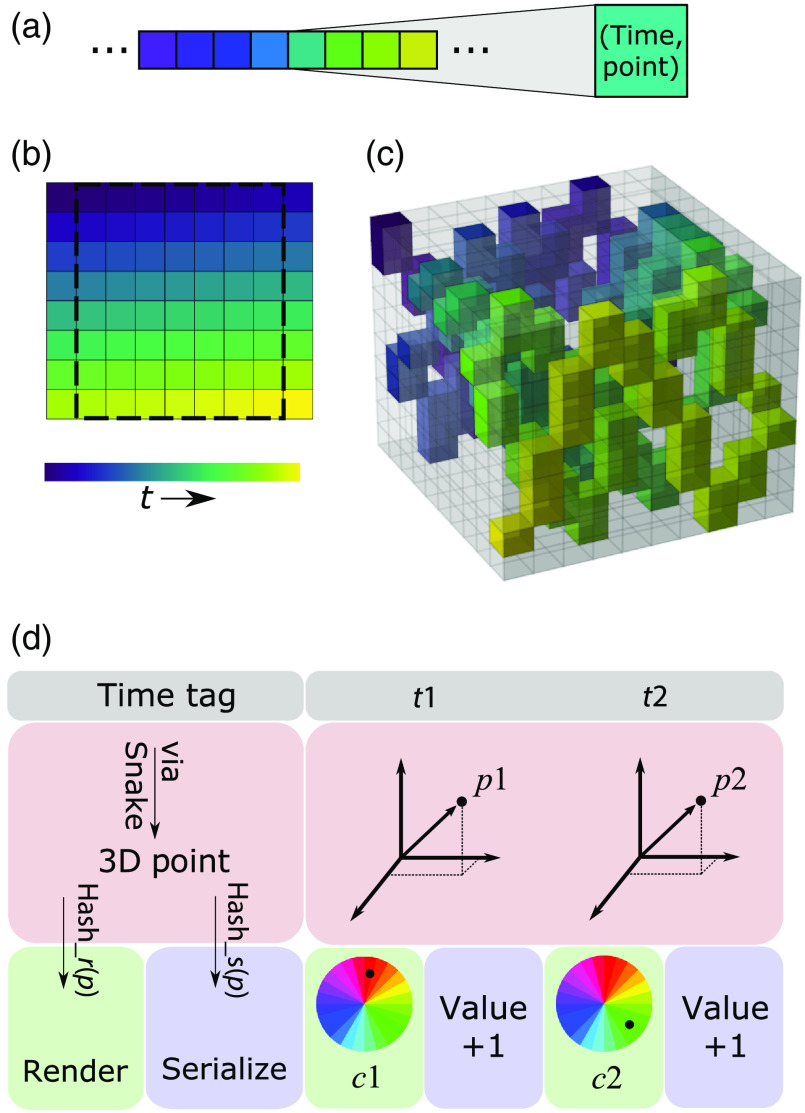
The allocation algorithm of rPySight. (a) A one-dimensional vector (“snake”) is generated from user inputs, modeling the number of pixels or voxels that the laser beam will traverse in the next rendered frame. Each cell in this vector has two properties—its end time and its associated coordinate, or point, in the rendered image or volume. The end time is specified in time units since the start of the experiment and signals the moment after which photons detected should not be allocated to this cell. The coloring marks the time axis. (b) A unidirectional 2D image with the mapping of the snake onto it. When imaging in 2D, the snake can be mapped to the plane almost directly. The only caveat lies at the edges of the image, where the resonant mirror is rotating and image deformation occurs. rPySight—similar to all other imaging software—discards a portion of these outer pixels due to these deformations so that the visible imaged is only composed of the pixels inside the square. (c) 3D mapping of the snake to the rendered volume. Some voxels are skipped due to the mismatch in frequencies between the galvanometric mirror and the TAG lens, but they will be sampled later because the scanning elements are not synchronized. (d) The computational pipeline that processes the time tags. Once a photon-based time tag is registered and sent to rPySight, it can be converted into a coordinate or point in 3D space using the snake-like structure explained above. This point is then sent to two different paths using a hashing function—the renderer assigns a color to that point based on the previous brightness of that voxel and on the spectral channel from which the event arrived, and the serialization process simply increments the value at that voxel because each spectral channel is independent of the other ones.

rPySight’s architecture is composed of three main parts. The first is a Python application that interfaces with the built-in application programming interface (API) of the TT. This module instantiates the TT with user-defined parameters and listens for events arriving to the designated channels. This polling loop, which samples at a minimum rate of 300 Hz, transmits the recorded event using inter-process communication protocols to the second rPySight module, the one responsible for assigning the detected photons to their voxel. This part of the library finds the appropriate coordinate by constructing a one-dimensional “snake,” illustrated in Figs. 2(a)–2(c). The snake is a projection of the trajectory that the scanned illumination laser beam will traverse in the sample. In the 2D case, it is a familiar raster pattern that takes into account the field fraction [i.e., the portion of the scanned path that will become the FOV displayed, i.e., pixels enclosed in the dashed-line box, Fig. 2(b)]. In 3D [Fig. 2(c)] this trajectory is not identical between volumes due to the resonant nature of the TAG lens, which means that it is unsynchronized with the rest of the scanners. The snake—built for every frame or volume on-the-fly—holds the mapping between the time of arrival to a specific pixel or voxel since the beginning of the current acquisition and the matching coordinate (2D or 3D) for photons arriving up to that moment. Then, for each detected photon, rPySight has to iterate over the snake and find the first time that the pre-computed pixel/voxel time exceeds this photon’s arrival time (computed from the beginning of the current frame or volume). The location of this occurrence (i.e., location along the snake path) is the coordinate to which the photon will be assigned. rPySight has several built-in mechanisms to render this process efficient and robust.

The third part of rPySight is responsible for rendering and data serialization. First, it receives a list of coordinates that house a photon. This list might contain duplicates, i.e., coordinates that were assigned with more than one event; therefore, the first task of this module is to increase the brightness of the coordinates that were populated with more than one photon, and it achieves that goal with a hashmap. Following that computation, the module sends a data buffer to the graphical processing unit (GPU) for rendering and a per-spectral-channel buffer to the disk for serialization and other post-processing applications.

Appendix C describes how to use rPySight during a photon counting experiment with and without a TAG lens, which provides continuous volumetric imaging of the sample.

### 2.4 Calcium Imaging Analysis and Statistical Testing

To detect and segment the active components, in the recording we used CaImAn,^11^ a calcium imaging analysis framework written in Python. Both datasets (analog and digital) were analyzed using identical parameters, and the output of the analysis—ΔF/F traces per active component—underwent peak detection using SciPy-based algorithms^19^ to mark all spike-like events in the recording.

The statistical tests we used are based on DABEST,^20^ an estimation statistics library that mainly uses bootstrap-oriented methods to compare two distributions. Importantly, this process focuses on the measured effect size rather than mere P-values; this is to illuminate the actual differences between the two tested datasets, eliminating cases with distributions that were very similar in absolute terms but turned out to be significantly different due to a large number of samples that was used. Importantly, the bootstrap process provides a confidence interval around the effect size, which allows for determining if the effect size, regardless of its magnitude, differs from zero or not. The distribution of bootstrapped values and this confidence interval are computed based on the mean differences between groups. Here, the effect size refers to the mean value of rPySight metrics “minus” the mean value of the same metric obtained during analog acquisition.

## 3 Results

### 3.1 rPySight Enables Calcium Imaging with Expected Improvement in Related Metrics

**Fig. 3:**
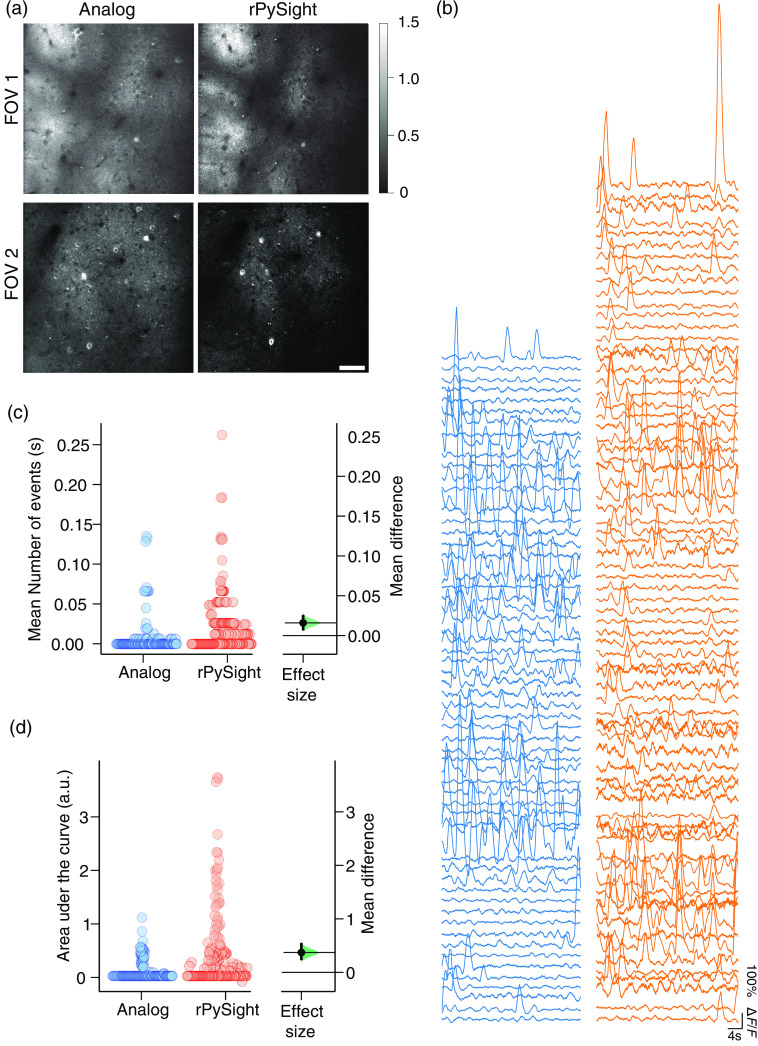
Calcium imaging using analog integration (blue) and digital photon-counting of three FOVs of a single Thy1-positive mouse. (a) Two representative FOVs from analog (left) and digital data acquisition, showing an average of 100 consecutive frames (data acquired at 30 pfs). The color bar marks the number of photons per pixel that were detected. Although for the digital image this number is accurate, the analog image was first normalized to the digital image’s scale to provide an accurate qualitative comparison. This normalization visually emphasizes the lower background levels of the photon counting image. (b) Exemplary ΔF/F traces for the two modalities calculated using CaImAn.^11^ The distinction between the relatively-quiet analog traces and the higher-amplitude digital ones, for the same FOV, is clearly visible. (c), (d) Summary statistics comparing analog and digital acquisition, showing a significant improvement in imaging conditions in favor of digital acquisition. The compared measured values were the mean number of spike-like events (c) and the mean area under the curve per spike-like event (d), and they were both significantly higher in the digital acquisition case. The effect size [right side of panes (d) and (c)] along with the corresponding confidence interval were computed around the mean difference between rPySight versus analog by bootstrap-coupled estimation using the methods introduced in Ref. 20, and the P-value was <0.001 for all comparisons, including median-based ones (not shown). The confidence intervals for the mean differences (vertical line and the entire distribution of bootstrapped estimates (green) are larger than zero, indicating the statistical significance of this comparison. Scale bar in (a) is 50  μm.

The importance of calcium and voltage imaging in the field is undeniable, as numerous labs rely on then to provide answers to countless questions in the areas of neural coding and encoding. We have previously demonstrated that photon-counting improves both calcium and voltage performance in these imaging modalities.^17^ Here, we exemplify that calcium imaging is improved when rPySight is used during *in vivo* recordings of calcium activity in the murine neocortex. During these experiments, each FOV was imaged twice—first using analog integration and then using the new digital setup. The acquisition parameters of each imaging modality were visually tuned for a maximal SNR before acquiring the data, which is summarized in Fig. 3. We acquired here three different fields in one animal, rendering bias to a particular condition very unlikely. In Fig. 3(a) 100-frame average images in two different FOVs are contrasted, after a normalization process during which the analog image was brought to the same scale as the digital one. The digital image’s brightness values accurately represent the number of photons detected per pixel, whereas in the analog image the value is an estimation. CaImAn^11^ detected traces for the second (bottom) FOV are displayed in Fig. 3(b).

To compare the two modalities, we pooled all three FOVs together. First, we note that the number of detected spike-like events in the digital recording is larger [0.01 to 0.026 spike-like events per second, Fig. 3(c)]. Second, the mean area under the curve (AUC) of the spike-like events in the digital recording was also larger [0.09 to 0.48 A.U., Fig. 3(d)], even though more of them were detected. Together with the fact that more active components were detected in the digital recording (145 to 119, components underwent manual filtration before being included in this count), these findings reiterate previously shown benefits of photon counting versus analog integration in calcium imaging experiments (for a detailed, cell-by-cell comparison please see Ref. 17). For the sake of completeness, we also note that classical Student’s t-test also found the measurements detailed above to be significantly different, with a P-value <0.0001.

### 3.2 rPySight Facilitates Advanced Multiphoton Imaging Modalities

**Fig. 4:**
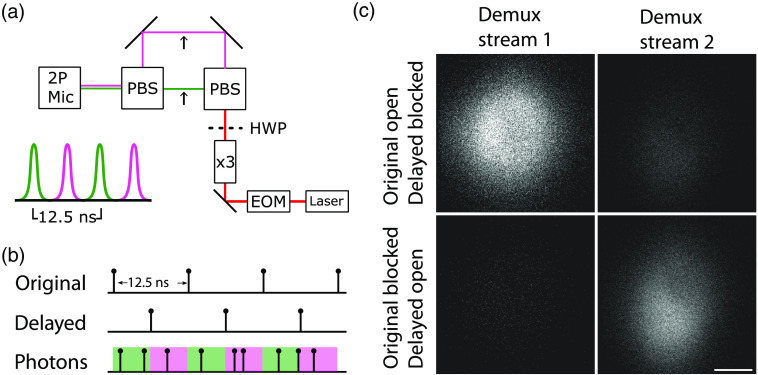
Real-time demultiplexing using rPySight. (a) Schematic of a simplified version that the laser path used to generate two pulse trains separated in time by half the laser inter-pulse period (top) and corresponding pulse train (bottom) for the original (green) and delayed (purple) paths. For the 80 MHz source used in this experiment, delay between each pulse train corresponds to 6.25 ns. (b) Schematics of events recorded by the TT. The original pulse train (top row), showing a typical 80 MHz signal, is duplicated and delayed internally (i.e., by the TT, middle row), which lets the TT interpret these two trains as the starting and closing signals for two alternating data streams, illustrated in green and purple in the bottom row. The arriving photons will be dynamically placed in one of the two demultiplexed event stream, which are rendered as separated channels according to their time of arrival since the last pulse. (c) Demultiplexing of photons emitted from a fluorescein bath imaged through the setup shown in (a) is demonstrated by alternatively blocking each beam path at the locations indicated by the arrows in (a). When the delayed beam path is blocked (top row), photons are detected only in the first demultiplexed stream (Demux stream 1). In turn, when the original beam path is blocked and the delayed path is open (bottom row), the opposite occurs and photons are only detected in the second demultiplexed stream (Demux stream 2), which corresponds to a 6.25 ns delay with respect to the original laser pulse emitted by the laser. Abbreviations: 2P-Mic, two-photon microscope; PBS, polarizing beam splitter; HWP, half-wave plate; × 3, three times beam expander; EOM, electro optical modulator. Scale bar is 100  μm.

**Fig. 5:**
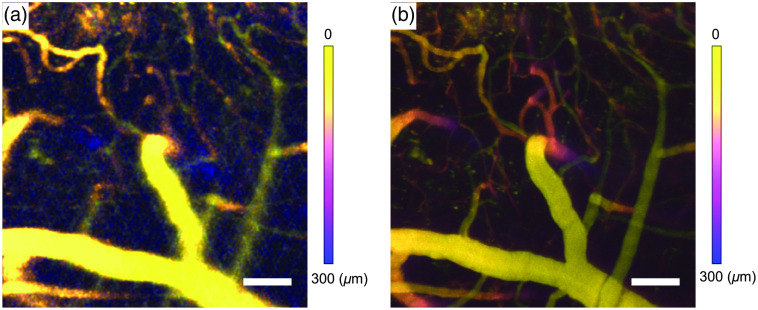
Real-time rapid continuous volumetric imaging in awake murine brain using rPySight (a) Depth-color-coded maximum z-projection of a real-time rendering of a $480\times 480\times 300\;{\mu m}^{3}$ volume continuously imaged at 30 volumes per second using a TAG lens. The temporal averaging window spans 550 frames, corresponding to 18.3 s. Following brightness normalization along the z axis, the image was filtered with a 3D Gaussian window using a $1.5\times 1.5\times 0.5\;{\mu m}^{3}$ kernel. Scale bar is 75  μm. (b) Depth-color-coded maximum z-projection of an offline rendering of the same volume using rPySight, summed across the entire recording (22,620 frames, corresponding to 754 s). Its improved signal-to-background ratio reveals finer micro-vascular features. Following brightness normalization along the z axis, the image was filtered with a 3D median filter of $1.5\times 1.5\times 2\;{\mu m}^{3}$. Scale bar is 75  μm. Both depth color codings are identical and were rendered using the Z-stack Depth Colorcode plugin of ImageJ.^21^

Figures 4 and 5 showcase two unique modalities that are difficult to implement in non-digital acquisition. The first (Fig. 4) is real-time demultiplexing of two or more photon streams into separate spectral channels. This is done by connecting the laser synchronization signal to one of the TT’s inputs and changing the demultiplex flag in the rPySight configuration file to true. The second modality is 3D continuous volumetric imaging, using a TAG lens, and is shown in Fig. 5. To demonstrate the demultiplexing capabilities, we built a two-arm setup before our 2PM in which one of the beams in the path is delayed by 6.25 ns [Fig. 4(a)]. The beam paths can be then alternatively blocked, and fluorescence emitted from a fluorescein bath is expected to be visible in only one of the demultiplexed data streams at the time.

Dividing the photons in real-time requires (in the case presented here) two laser synchronization signals to be connected to the input ports of the TT, one for each data stream. Because our excitation laser only emits a single synchronization signal [Chameleon Discovery NX TPC, Coherent Inc., represented Fig. 4(a)], the TT automatically duplicates this signal and shifts it by 6.25 ns to receive two independent pulse trains. These then serve as a gating mechanism for the TT—when the first one arrives, the gate (or window) for the first channel opens while the gate for the second channel closes, and when the second pulse arrives after 6.25 ns, the gate for the first channel closes and the gate for the second opens. The TT is able to allocate the photons arriving during each of these periods into their own data stream, which is broadcasted independently to the computer. Our experiment showed that rPySight correctly assigned these demultiplexed streams into separate channels that became almost dark with the corresponding beam path was blocked [Fig. 4(c)]. The minor amount of cross-talk that remains and is visible in Figs. 4(c) and 4(d) has to do with the two beams not being exactly 6.25 ns apart in our optical setup.

The two laser pulse trains incur some post-processing penalty on rPySight due to their high rate. To reduce it as much as possible, we deploy a conditional filter with these channels and the PMT channels. The filter waits for a photon to arrive at one of the data channels, and only when an event is registered does it also register the last incoming laser pulse (which was detected but not included in a data stream to avoid processing). Recalling that a low probability of fluorophore excitation per laser pulse ($P_{\text{pulse}}<0.1$) should be retained to prevent an effective broadening of the point spread function,^22^ this filtration step matches the recorded event rate to the rate of photon detection, rather than to the much higher doubled laser pulse repetition rate.

As noted, rPySight also facilitates rapid 3D imaging. A TAG lens was used for continuous imaging of a $480\times 480\times 300\;\mu m^{3}$ volume at an imaging rate of 30 volumes per second. The volumetric image shown in Fig. 5(a) is a maximum Z-projection of the volume rendered by our application during an *in vivo* recording of the vascular diameter in a Texas Red-injected mouse. The real-time rendering is sufficiently detailed to allow the experimenter to choose a volume of view centered on a penetrating artery, and an imaging depth that retains simultaneous tracking of its respective pial artery. Smaller features are buried in the background due to a short temporal averaging window (550 frames, corresponding to 18.3 s; notice that volumetric imaging is inherently reducing photon flux from each plane; hence the longer average is needed to display a volumetric image compared with a single plane imaged at 30 fps as shown in Fig. 1(c) and sub-optimal line-shift correction. These parameters can both be corrected offline. Indeed, when summing the entire 754 s long recording over time as depicted in Fig. 5(b), the improved signal-to-background ratio of the respective z-projection reveals finer details such as pial veins, arterioles, and capillary vessels, as well as the deeper portions of the imaged penetrating arteries.

## 4 Discussion

This work introduced an innovative software geared to allowing researchers to easily incorporate photon counting and perform highly-tailored experiments using this approach. The presented software focuses on the ability to render in real time the experiment being conducted, which improves on our past work and further facilitates these kind of experiments. We also updated the hardware used to a more user-friendly (from the programming and usage point of view) and high-performance fast digitizer. We also showcased the advantages of photon counting for multiphoton imaging by demonstrating its effectiveness in a variety of use-cases, some of which are exclusive to photon counting. The improvements demonstrated here were made possible by introducing a fast digitizer to the acquisition hardware and using the open-source software solution that was developed for this work,^18^ and they require very little technical expertise or pre-existing knowledge. In addition, the post-processing step that was a hard requirement in our previous work^17^ and hurt the user experience is no longer required (although it improves results as the off-line processing is tailored to better handle artifacts emerging from timing discrepancies between scanning elements) providing the experimenter with a (potentially 3D) view of their dataset during the live experiment.

A shortcoming of our application is its hardware requirements. rPySight requires a GPU for it to function properly and in a performant manner. Furthermore, processing long lists of time tags also puts a toll on the central processing unit, and together these hardware requirements demand that users run the software on a capable workstation. It is worth noting that low photon fluxes, at rates of up to a few hundred thousand events per second, which are typical when imaging deep in the cortex, are easily parsed and displayed by rPySight even on low-end machines. In addition, planned firmware and software upgrades for the TT itself will also reduce the computational constraints on some of the more costly rPySight operations, such as demultiplexing, which could be migrated to the device field-programable gated array.

Looking into the very near future, we foresee the introduction of fast volumetric imaging, as enabled by time-tagged photon counting, as playing a crucial role in the quest to uncover the hemodynamic transform function as derived from populations of neurons and multiple blood vessels. There are several reasons for pushing neurovascular studies into a “volumetric” modality. In one hand, the inherent flow dynamics across vascular trees dictates a high-correlation between observed dynamics in topologically-close vessel segments. On the other hand, there is a high dependency between large amplitude flow changes, which manifest as propagation waves, and locally observed dynamics. Being able to capture this rich set of interactions can only be done while imaging, for example, across the depth of the cortex. Further, one needs to simultaneously capture the cellular dynamics, being from neurons of different populations, astrocytes, or mural cells, to be able to establish with high-fidelity, a correlation between vascular and cellular dynamics. As we exemplified here, even when imaging neuronal dynamics in a single diffraction limited plane at 30 fps, cellular dynamics are better observed through photon-counting rather than analog integration. It follows from this that the more challenging photon-deprived conditions present in volumetric imaging (i.e., expanding the same FOV across multiple imaging planes) might only be addressable when implementing photon counting approaches such as the one described here.

## 5 Appendix A: Animal Preparations

For the *in vivo* experiment, adult male mice of the C57BL/6J-Tg (Thy1-GCaMP6f) GP5.5Dkim/J line, which are known to have fluorescent excitatory neurons in layers II/III and V of their cortex, were used. Their genotype was verified by polymerase chain reaction analysis of tail DNA. Mice were housed in Tel Aviv University’s Green Building vivarium under standard conditions.

For the optical window, following Kim et al.,^23^ mice were anesthetized using isoflurane (4% for induction and 1% to 2% during surgery). To minimize inflammation and brain edema, carprofen (5 to 10  mg/kg) and dexamethasone (2  mg/kg) were injected subcutaneously prior to surgical incision. Mice were kept at 37°C using a heating pad (CWE, TC-1000 Mouse). Skin was removed, and the skull surface was cleaned with a scalpel while continuously perfusing artificial cerebrospinal fluid (ASCF) over the surgical area. Next, craniotomy matched in size and shape to the outline of the crystal skull glass (Labmaker, Crystal Skull-R10) implant was performed. To make the craniotomy, we first created a shallow groove along the perimeter of the crystal skull glass using a 0.7-mm-diameter drill (Henry Schein, #9998049). We used a 0.5-mm-diameter drill (Henry Schein, #9990862) to deepen the groove and disconnect the trapezoidal-shaped bone piece from the surrounding skull. We perfused ACSF solution throughout the drilling. Once the bone piece was disconnected from the skull, we removed the overlying bone piece with forceps while positioning the glass implant. The dura was left intact.

Once the Crystal Skull implant was in its final position, we dried its edges and glued the window to the skull with UV-light-cured adhesive (UV glue; Loctite, 4305). To allow for head fixation during optical brain imaging, we installed a stainless-steel head plate using UV-light-cured adhesive and sealed the edges using Clear Ortho-Jet dental cement (Lang Dental Manufacturing, Ortho-Jet Package). After curing the glue and cement, we cleaned the surface of the window and covered it with a fast cure platinum-catalyzed silicone (Smooth-On Inc., Ecoflex™ 00-35 FAST) to protect the window when mice are not imaged. We transferred the mouse to a recovery cage and placed it on a heating pad until it awoke. We then returned the mouse to its home cage and provided food and water on the cage floor without use of a food hopper, to prevent damaging of the crystal skull window. We subcutaneously administered the mice carprofen (5 to 10  mg/kg) for the first 3 days after surgery. For the first 7 days after surgery, we checked all mice daily for signs of distress. Mice were allowed to recover for 3 weeks before imaging.

## 6 Appendix B: Detailed Overview of the Optical Setup

Our 2PM is a slightly modified version of the Movable Objective Microscope (MOM, Sutter Instrument Company). The pulsed laser source is a Coherent Chameleon Discovery NX TPC emitting 930 nm pulses at 80 MHz. The internal dispersion compensation was used to increase the measured brightness of the sample. The beam first goes through a magnifier (GBE03-B, Thorlabs, Inc.) and a custom × 1.5 telescope to collimate the spot and to magnify it to the expected waist diameter at the entrance to the MOM. Before meeting the resonant-galvo scanners (RESSCAN-MOM, 8 kHz), the beam optionally passes the TAG lens (TAG lens 2.5β, TAG Optics) with a 4f relay system (AC254-045-B, AC254-060-B, Thorlabs, Inc.) that images the ultrasonic lens, resonating at about 189 kHz, to the scanners.

The beam bounces off the scanners through the scan and tube lenses (50 mm EFL, Leica Microsystems and 200 mm EFL CFI #93020, Nikon Instruments) and through a dichroic mirror (BrightLine FF735-Di01-25×36, Semrock) before finally reaching a 10×0.6 numerical aperture objective lens (XLPLN10XSVMP, Olympus Corporation), which both excites the sample and collects the returning fluorescent light. A low magnification objective lens is necessary to obtain an axial range of 300  μm from the TAG lens, the effective axial range of which drops proportionally to the squared magnification of the objective lens. These returning photons are deflected by the dichroic mirror into the secondary dichroic mirror (565dcxr, Chroma Technology Corporation), which splits the photons into green and red ones. The green photons pass through a bandpass filter (525/70-2P, Chroma Technology Corporation) before being collected by a PMT (H10770PA-40SEL, Hamamatsu Photonics K.K.). The red channel photons also pass through a bandpass filter (BrightLine FF01-625/90-25, 25×25, Semrock) before being collected by an identical PMT.

The green channel’s signal is amplified using a fast preamplifier (TA1000B-100-50, FAST ComTec GmbH) while the red channel’s photon are amplified by a 200 MHz preamplifier ((DHCPA-100, FEMTO Messtechnik GmbH) and discriminated by a fast discriminator (TD2000, FAST ComTec GmbH). Both signals are then routed to the TimeTagger Ultra (Swabian Instruments GmbH), which serves as a digitizer and transmits the digital photon stream to the acquisition computer via a USB 3.0 interface. The TT receives other analog inputs as well, namely the line and frame synchronization signals that the microscope control software (ScanImage 2021, Vidrio Technologies LLC^10^) outputs via its breakout boxes (BNC 2090 connected to a FlexRIO PXIe-1073 with a 5734 adapter module sampling at 120 MHz, National Instruments). The microscope itself—its scanners, shutter, and power modulation—are all controlled by the mentioned control software (ScanImage).

## 7 Appendix C: Capturing and Visualizing Real-Time Photon Counting 2D/3D Data—A Protocol

### 7.1 C.1 Preparations

The following procedures should be done on the computer that controls the 2PM with its dedicated software and hardware.

1. Download rPySight from the GitHub repository: https://github.com/PBLab/rpysight. You may install the binary from the “Releases” section (preferred) or download the source and compile it yourself. In the latter case, you’ll also need a Rust compiler, which can be obtained from Ref. 24.
2. Install the TT on your computer by following their guide.^25^ Make sure to follow the Python section to verify that you are able to communicate with the TT via its Python API.
3. Download the call timetagger.py file from the base repository and run it. It should succeed in importing the TimeTagger library, which indicates that the installation was successful.
4. Run rPySight. Open a terminal and run rPySight by typing <PATH-TO-EXE>\cli.exe. The process should hang indefinitely while rPySight waits for a line or frame signal that will start its rendering process. If the process immediately crashes then the installation procedure of either the TT or rPySight was unsuccessful. If the next two steps of this tutorial are not working as intended, please open an issue in rPySight’s repository; otherwise, try to re-install the TT.
5. Connect synchronization signals from the microscope’s control boards to the TT’s inputs similar to Fig. 1. In a minimal experimental setup, the required signals are a single PMT, preferably after preamplification if the preamp is fast enough (at least 200 MHz), and the line synchronization signal, i.e., a cue that signifies that a new line has started. Other optional cables include a frame synchronization signal, the laser’s synchronization signal, the TAG lens synchronization pulses, and more PMTs.

**NOTE:** If you have never inspected the signals emitted by your scanners and PMTs, it might be better to first connect them to an oscilloscope to check their amplitude and polarity. Signals stronger than ±5  V or 50 Ω impedance will harm the TT, while a weak signal might not be detected reliably by the 50 Ω-terminated inputs.

6. Configure the TT. Start the TT’s native software and initialize the device. Access the settings menu (gray cogwheel), and set the active channels of the device based on the connections that you made in the previous step. Set the threshold value per channel to a value that is far enough from the base noise level. Setting the right threshold is usually optimized empirically.
7. Test the incoming signals. Start the acquisition system to activate the synchronization signals and turn on the PMT to make sure that some photons are detected and pre-amplified before arriving to the TT. Next, start a TT measurement of type “Counter” for each connected channel. You should now see the two data streams on your screen. Assert that the event rate is as expected—a resonant scanner should emit synchronization signals at its nominal frequency, whereas a PMT should emit photons at rates ranging from a few thousands of events per second to millions of them, depending on the light level.
8. If the event rates are at a sufficient pace, turn off the measurement and disconnect the TT using the cogwheel menu. You are now ready to run rPySight.

**NOTE:** rPySight cannot work while the TT is connected to the web user interface. You must first shut down the TT from the settings menu before starting rPySight yet leave the server running [as shown in Fig 1(c)]

### 7.2 C.2 Acquiring Data

1. Prepare your setup for data acquisition as done regularly for your experiments.
2. Prepare the rPySight configuration file. You may use the pre-made file available in rPySight’s repository and modify it to fit your setup:
  • Change the input channels and thresholds to the values you set in the web-based user interface of the TT. Negative channel values (e.g. “−2”) mean that the channel should detect the falling edge of a signal.
  • Set the number of rows and columns of the image to match the values in your microscope acquisition software.
  • If you have a TAG lens or any other remote focusing device that provides a synchronization signal for a specific phase of the scan, make sure to enter its nominal frequency and set the number of planes to some even value larger than one.
  • Change the value in the filename field to make sure data is recorded to the expected folder.
3. Run rPySight by issuing the command cli.exe <PATH-TO-CONFIG.toml> in your terminal. After a few seconds, a blank window should pop up, and it will remain blank until the first line or frame signal is received.
4. Start the other auxiliary devices, such as the TAG lens or a behavioral camera.
5. Start the acquisition and observe the rPySight window. Assuming that everything was set up correctly, you should see the 2D or 3D image on your display.
6. Assert that data are being recorded to the folder you set in the configuration file. You should see a .ttbin file created by the TT and an .arrow_stream file that saves the coordinates of the displayed pixels.

### 7.3 C.3 Data Inspection and Post-Processing

The data that were collected and displayed by rPySight may be re-used following the experiment in a few ways:

• ttbin files can be replayed using rPySight with different parameters, such as different averaging, more or less columns in the rendered image, and with or without some of the data channels. To replay a file, the filename field in the configuration file should point to an existing file, and the replay existing field should be set to true.

**NOTE:** Re-running an experiment also overwrites the .arrow_stream file, so make sure to backup the previous one if you wish to keep it.

• rPySight’s .arrow_stream files let you re-render the images or volumes in a rendering program of your choice. An example of such a feat can be found in rPySight’s repository.

## Code, Data, and Materials Availability

rPySight is an open-source, GPLv3 application available as a code repository on GitHub: https://github.com/PBLab/rpysight. The data captured for this work is available from the authors upon request.
